# Effects of Flag Leaf and Number of Vegetative Ramets on Sexual Reproductive Performance in the Clonal Grass *Leymus chinensis*

**DOI:** 10.3389/fpls.2020.534278

**Published:** 2020-10-30

**Authors:** Jian Guo, Haiyan Li, Chan Zhou, Yunfei Yang

**Affiliations:** ^1^Key Laboratory of Vegetation Ecology, Ministry of Education, Institute of Grassland Science, Northeast Normal University, Changchun, China; ^2^School of Life Sciences, Liaoning University, Shenyang, China

**Keywords:** flag leaf, perennial herb, resource allocation, resource translocation, sexual reproduction, tillering node, vegetative ramet

## Abstract

Sexual reproduction is vital for population adaptation in clonal plants. The flag leaf is considered to be the primary contributor to sexual reproduction in cereal crops, and there is no unified conclusion on the effect of the number of vegetative ramets on grain yield. However, what effects of the flag leaf and the number of vegetative ramets on sexual reproductive performance of clonal grasses are largely unknown. To test this, under field natural conditions, we grew the rhizomatous grass *Leymus chinensis* in a homogeneous environment and conducted studies concerning the growth, reproduction and physiology of reproductive ramets in clonal populations. We measured the growth characteristics of different aged leaves, dynamically measured the net photosynthetic rate of different aged leaves and organ biomass, measured the sexual reproductive characteristics of reproductive ramets that had different numbers of connecting vegetative ramets, and performed isotope (^15^N) labeling of ramet pairs at the seed-filling stage. In *L. chinensis* clonal populations, from the heading stage, the photosynthetic contribution of the functional leaves to seed production was much greater than that of the flag leaf; the photosynthetic capacity of both the functional leaves and the flag leaf all gradually declined. Vegetative ramets translocated their own resources to the connected reproductive ramets, and a large proportion of translocated resources were allocated to the leaf and stem to sustain life activities; increase in the number of connecting vegetative ramets increased floret number, seed number, seed-setting rate, inflorescence biomass, seed biomass, and reproductive allocation of reproductive ramets, and these parameters significantly and positively correlated with the biomass of connecting vegetative ramets. We conclude that the functional leaf rather than the flag leaf of *L. chinensis* is the primary contributor to seed production. Reproductive ramets adopt a strategy of growth first and reproduction later to allocate the translocated resources between the organs, but vegetative ramets are very advantageous for sexual reproduction under the tillering node connection form in *L. chinensis*. Overall, our study implies that vegetative ramets not only play an important role in the spatial expansion but also in the sexual reproduction of clonal plant populations.

## Introduction

Reproduction is an important link in the entire life history of plants, which is related to the formation, development, and evolution of plant populations. Many higher plant species are clonal organisms that have the capacity to propagate vegetatively by means of specialized organs such as rhizomes, stolons, bulbils, and rooting buds or reproduce sexually through seeds (Richards, 1986). Within a clonal plant, the offspring derived from vegetative propagation are specifically called “ramets” (Harper, 1977); they are physiologically potentially independent but share an identical genetic makeup. In contrast, the offspring generated by sexual reproduction are genetically diverse and better able to adapt to heterogeneous or changing environments (e.g., Maynard Smith, 1971), and they can spread over longer distances (Eckert, 2001). Under natural conditions, some clonal plant populations mainly rely on vegetative propagation to achieve recruitment and regeneration with infrequent recruitment from seeds (Cook, 1985; Eriksson, 1989, 1992), but sexual reproduction is vital for the long-term development of clonal populations.

There are two types of ramets widely distributed in clonal plant populations: vegetative ramets and reproductive ramets (Harper, 1977; Wang et al., 2017). Vegetative ramets refer to ramets that do not produce flowers and solely conduct vegetative growth, while reproductive ramets refer to ramets that enable sexual reproduction, flowering, and seed setting. Once a reproductive ramet initiates reproductive machinery via growth, a strong source-sink relationship is formed between the organs (Pan, 2012). Inflorescences are forming seeds and are a sink for accumulating nutrients; leaves can carry out photosynthesis and are a source for producing nutrients (Chang and Zhu, 2017; Burnett, 2019). In gramineous plants, the first leaf below the inflorescence is usually called the flag leaf (the last leaf to emerge), and the leaves at other positions of a plant are called functional leaves. Many studies concerning cereal crops have shown that the flag leaf is the primary contributor to sexual reproduction (Walpole and Morgan, 1972; Yoshida, 1972; Araus and Tapia, 1987). For example, Li et al. (1998) found that the upper leaves of the rice plant, particularly the flag leaf, provided more than 50% of the dry matter for grain filling. Lupton (1972) estimated by modeling approaches that at least 50% of the grain carbohydrate was derived from the flag leaf. However, the extent to which the flag leaf of clonal grass contributes to seed production during sexual reproduction is still unclear.

Ramets are usually connected to each other in clonal gramineous plants. Except for rhizome or stolon connections, tillering node connection is also a very common connection form between ramets. Under natural conditions, vegetative ramets may grow on the unelongated basal internodes of reproductive ramets, in which case the two types of ramets are interconnected through tillering nodes. Surprisingly, direct tests of the effect of vegetative ramets connected to tillering nodes on reproductive ramets appear to have focused mainly on cereal crops. Vegetative ramets can compete with reproductive ramets for solar energy, carbohydrates, and mineral nutrients, for example, nitrogen in rice (Nuruzzaman et al., 2000); thus, an increase in vegetative ramets may cause fewer nutrients to be directed to grain production. However, Ao et al. (2010) found that an increase in the number of vegetative ramets did not reduce the grain yield of rice. Similarly, many studies concerning wheat have also observed inconsistencies in the effects of vegetative ramets on grain production (Thorne and Wood, 1987; Zhou et al., 2007). However, it is not well known what effects of the number of vegetative ramets connected to tillering nodes on seed production in clonal grasses.

*Leymus chinensis*, a perennial rhizomatous forage grass, is often regarded as a constructive and dominant species across eastern areas of the Eurasian steppe (Zhu, 2004). Under natural conditions, *L. chinensis* primarily depends on vegetative propagation of rhizomes and tillering nodes to achieve population renewal and spatial expansion (Yang et al., 1995), and its capacity for sexual reproduction is rather weak. It has been reported that seed-setting rate is less than 25% on the average in natural grasslands, but varies greatly between individual ramets (Wang, 1998). Because of economical and ecological significance, *L. chinensis* has received considerable attention (Wang, 2001; Gao et al., 2014; Zhou et al., 2014; Yuan et al., 2016). Many studies have shown that seed production of *L. chinensis* can be influenced by external ecological factors, such as climate (Yang et al., 2000; Wang et al., 2003), water use (Song et al., 2003), nutrient uptake (Chen J.S. et al., 2013), and disturbance by human being and animals (Wang, 2000). However, how vegetative ramets within the clone affect seed production of *L. chinensis* has less been studied.

Here, we grew *L. chinensis* in a field homogeneous environment and measured the growth characteristics of different aged leaves of reproductive ramets, dynamically measured the net photosynthetic rate of different aged leaves and organ biomass, determined the phenotypic characteristics of reproductive ramets which had different numbers (1, 2, 3, ≥4) of connecting vegetative ramets. We labeled the vegetative ramets with isotope (^15^N) at the seed-filling stage to verify whether vegetative ramets translocate resources toward the connected reproductive ramets. The objectives of our study were (1) to explore the contribution of the flag leaf to seed production during sexual reproduction and (2) to assess the effect of the number of vegetative ramets connected to tillering nodes on sexual reproductive performance. Here, we hypothesize that during sexual reproduction, (1) the photosynthesis of flag leaves will contribute the most to seed production, and (2) increase in the number of vegetative ramets connected to tillering nodes will reduce sexual reproductive performance.

## Materials and Methods

### Study Area

The study was conducted at the Grassland Ecological Research Station, Northeast Normal University, Jilin Province, China (44°38′ N, 123°41′ E). The study site is located in the southern region of the Songnen Plain, where the climate is semihumid and semiarid, with hot, rainy monsoonal summers and cold, arid winters. The mean annual temperature is 4.6–6.4°C. The mean annual precipitation is 300–450 mm, mostly falling in the growing season, and the annual evaporation is 1200–1400 mm. The growing season with a frost-free period is approximately 130–165 days (Li et al., 2018; Guo et al., 2020).

### Study Species

*Leymus chinensis*, a perennial forage grass, has high nutritional value and good palatability. *L. chinensis* has strong ecological adaptability and tolerance to drought, salt-alkaline, and low-temperature conditions (Bai et al., 2004; Jin et al., 2008; Chen S. et al., 2013); thus, it often forms *L. chinensis* steppes and meadows as a dominant species. It is widely distributed in the eastern part of the Eurasian steppe zone, including the southern and central areas of Russia, the People’s Republic of Mongolia, the Inner Mongolian Plateau of China, and the Northeast China Plain (Kuo, 1987). The clonal ramets of *L. chinensis* are interconnected via rhizomes or tillering nodes (Guo et al., 2020). It is common for *L. chinensis* to have one reproductive ramet and different numbers of vegetative ramets on one tillering node. On the Songnen Plain, *L. chinensis* usually begins returning green in early April, heading in mid-to-late May, flowering in June, and maturing in mid-July (Zhu, 2004).

### Experimental Platform

A total of 30 experimental plots of *L. chinensis* were established at the beginning of May 2015. Adjacent plots were at least 2 m apart, and the area of each plot (2 m × 2 m) was 4 m^2^. Vegetative ramets of *L. chinensis* of a similar size (ramet height: 20.9 ± 0.3 cm, mean ± SE) were sampled from the same natural clone in the study area, and then nine ramets were transplanted into each plot with rows 0.5 m apart and 0.5 m between ramets. Ramets were only watered in the early stage of transplanting to ensure survival. During the entire experimental period, the plots were just weeded regularly and without any management of irrigation and fertilization, the ramets were not influenced by any insect pests or diseases. The experimental site was originally agricultural land. The soil type is sandy loam (Li et al., 2001; Guo et al., 2020; Yuan et al., 2020). The soil of the top 20-cm-thick soil layer was homogenous, and the total organic C content, total N content and total P content were 6.23 ± 0.55 g kg^–1^, 1.01 ± 0.04 g kg^–1^, and 0.74 ± 0.02 g kg^–1^, respectively. The pH was 8.37 ± 0.03, and the electrical conductivity was 70.85 ± 2.61 μS cm^–1^.

In 2015, the transplanted ramets of *L. chinensis* in each experimental plot only conducted vegetative propagation, and there was no reproductive ramet in the *L. chinensis* population. In 2016, a few reproductive ramets appeared in several experimental plots. In 2017, there were many reproductive ramets in each experimental plot. Therefore, this study started from the third year after the establishment of experimental plots. The preliminary investigation showed that approximately 93.3% of reproductive ramets had three leaves, and only 6.7% had four leaves in experimental plots. Thus, reproductive ramets with three leaves were chosen for all experiments in this study.

### Leaf Growth Characteristics, Photosynthetic Rate and Organ Biomass

To assess the effects of growth time on the leaf net photosynthetic rate and organ biomass of reproductive ramets, periodic measurements and samplings were conducted. A total of 10 plots were randomly selected from 30 plots to mark the samples on May 20, 2017. Twenty-two reproductive ramets that were healthy and uniform in height and whose inflorescences were fully exposed 2 cm away from the flag leaves were randomly selected and marked in each plot. One of 22 ramets in each plot was used as a fixed sample ramet to measure leaf photosynthetic rate, and the remaining 21 ramets were used to measure organ biomass.

At the early heading stage of *L. chinensis* populations, the net photosynthetic rate of all aged leaves on each marked ramet was measured every 7 days using a LI-6400 portable photosynthesis system (Li-Cor Biosciences, Lincoln, NE, United States) under the conditions of saturating light intensity (1500 μmol m^–2^ s^–1^) and fixed CO_2_ concentration (400 μmol mol^–1^). The measurements started on May 26 and ended on July 7 when the leaves turned distinctly yellow. According to the birth order of the leaves, the leaves were recorded as 1a-leaf, 2a-leaf, and 3a-leaf in order from the base to the top of the reproductive ramet. The 1a-leaf and 2a-leaf were functional leaves, and the 3a-leaf was the flag leaf.

The measurements of organ biomass were conducted simultaneously with photosynthesis measurements. For each measurement, only the aboveground part of the ramet was harvested, and a total of three reproductive ramets were randomly collected from the marked ramets with tags in each plot and dried to a constant weight at 65°C. Leaf biomass, stem biomass and inflorescence biomass were measured. All leaves of each ramet at the 5th measurement on June 23 (at the seed-filling stage, the most critical stage of sexual reproduction) were scanned on a flatbed scanner (CanoScan LiDE 110, Canon, Japan) to calculate the leaf area.

### Effects of the Number of Vegetative Ramets Connected to Tillering Nodes on Sexual Reproductive Performance

**FIGURE 1:**
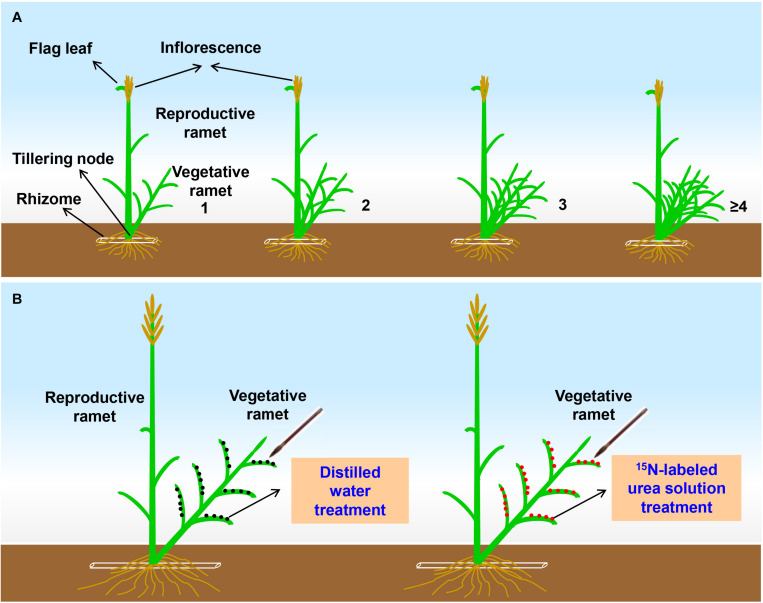
**(A)** Schematic representation of the tagging experimental design at the early heading stage of *Leymus chinensis*. The Arabic numerals represent the number of vegetative ramets connected to a tillering node of the reproductive ramet. In each gradient, inflorescence top of the reproductive ramet reaches approximately 2 cm over the flag leaf sheath. **(B)** Schematic representation of the stable-isotope (^15^N) labeling experimental design at the seed-filling stage of *Leymus chinensis*. Each ramet pair consists of one reproductive ramet and one vegetative ramet connected by a tillering node (which refers to unelongated basal internode of the ramet).

To assess the effects of the number of vegetative ramets connected to tillering nodes on sexual reproductive performance in a field homogeneous environment, grading samplings were conducted in both 2017 and 2018. First, at the early heading stage of *L. chinensis* each year, we used colored tags to mark those reproductive ramets whose inflorescence top reached approximately 2 cm over the flag leaf sheath and that were connected to different numbers (1, 2, 3, and ≥4) of vegetative ramets by tillering nodes at the edge of each plot (Figure 1A). There was only one reproductive ramet per gradient in each plot. Five plots were abandoned because some gradients were not found. In fact, the reproductive ramets that were connected to zero vegetative ramets via tillering nodes exist at the edge of few experimental plots, due to its extremely low numbers did not meet the requirement sample size for experiment, so it was not taken into account in this study.

At the seed-maturing stage, only the aboveground parts of both marked reproductive ramets and their connected vegetative ramets were harvested together. The inflorescence length was measured, and the floret number and seed number were counted. Inflorescence biomass, seed biomass, reproductive ramet biomass, leaf biomass, and total biomass of vegetative ramets were measured after drying in an oven at 65°C for 48 h.

### Stable-Isotope Labeling

To verify whether vegetative ramets translocate their own resources to the connected reproductive ramets during sexual reproduction, *in situ* labeling of vegetative ramets with ^15^N by foliar brushing (Putz et al., 2011; Wang et al., 2017) was carried out at the seed-filling stage of *L. chinensis* in late June of both 2017 and 2018. Four plots were randomly selected for ^15^N labeling each year. Two ramet pairs with similar sizes (one ramet pair for the ^15^N labeling treatment and another for the control treatment) were randomly chosen at the edge of each plot, and each ramet pair consisted of one reproductive ramet and one vegetative ramet connected by a tillering node (Figure 1B). Two ramet pairs were at least 50 cm apart. A ^15^N labeling experiment was conducted in the following year in randomly selected remaining plots to avoid cross-contamination.

The ^15^N labeling experiment was performed on consecutive sunny days so that the ^15^N label on the leaves of ramets would not be washed into the soil. Before labeling, the soil surface was covered with plastic film to prevent polluting soil, and the reproductive ramet of each ramet pair was kept in a plastic film cylinder sleeves to avoid contamination. One milliliter of ^15^N-labeled urea (made at Shanghai Research Institute of Chemical Industry, China) solution with a urea concentration of 0.02 g ml^–1^ and a ^15^N abundance of 5.18% was applied to all leaves of the vegetative ramet of each ramet pair (Figure 1B) following the protocol from Guo et al. (2020). Labeling was performed once a day for 3 days. An equal volume of distilled water was used instead of the ^15^N-labeled urea solution in the control treatment (Figure 1B).

On the 5th day of ^15^N labeling, the aboveground reproductive ramets both in the labeling treatment and control treatment in each plot were harvested and separated into three parts, namely, the inflorescence, stem, and leaf. Each plant tissue was de-enzymed at 105°C for 30 min and then dried at 65°C for 48 h. We then measured the dry mass of each tissue and ground the samples to a fine powder in a ball mill (MM 400 Retsch, Germany). For each sample, approximately 3 mg of solid powder was tightly wrapped in a capsule and then directly evaluated with an Isoprime 100 isotope ratio mass spectrometer (Elementar, Langenselbold, Germany) coupled to a vario EL cube elemental analyzer (Elementar, Langenselbold, Germany). We measured N content and δ^15^N. The δ^15^N was expressed as follows:

(1) $$\delta^{15}\,\text{N}\,\left(‰\right)=\left(R_{\text{sample}}/R_{\text{standard}}-1\right)\times 1000$$

where *R*_sample_ and *R*_standard_ represent the ^15^N/^14^N in a sample and ^15^N/^14^N in atmospheric N_2_, respectively. The ^15^N/^14^N in atmospheric N_2_ is constant at 0.00368.

The amount of translocated ^15^N from the labeled vegetative ramet toward each tissue of the unlabeled reproductive ramet was calculated by the following equations:

(2) $$F\left(\%\right)=\left[R_{\text{sample}}/\left(R_{\text{sample}}+1\right)\right]\times 100$$

(3) $${\text{Translocated}}^{15}\,N\,\mathrm{amount}=W_{\text{dry}-\text{labeling}}\times B_{\text{labeling}}\times \left(F_{\text{labeling}}-F_{\text{control}}\right)$$

where *W*_dry_ and *B* represent dry mass and nitrogen content per unit mass of a sample in the labeling treatment, respectively. *F*_labeling_ and *F*_control_ represent the *F*-ratio of a sample in the labeling treatment and control treatment, respectively.

### Statistical Analysis

All data analyses were carried out using SPSS 22.0 statistical software (SPSS Inc., Chicago, IL, United States). Data normality was tested with the Kolmogorov–Smirnov test, and variance homogeneity was tested with Levene’s test. The significance level was set at *P* < 0.05.

The length, width and area of flag leaves and functional leaves were calculated by WinFOLIA Pro 2011 software (Regent Instruments, Québec city, QC, Canada). One-way ANOVA was used to assess the effects of leaf age on the leaf length, width and area of reproductive ramets; to examine the effect of organ on the amount of translocated ^15^N; and to examine the effects of the number of connected vegetative ramets on sexual reproductive performance. Significant differences in the means were compared with Duncan’s multiple-range test. An independent-samples *t*-test was used to test the differences in leaf δ^15^N, stem δ^15^N, and inflorescence δ^15^N between the ^15^N labeling and control treatments. Sexual reproductive performance consisted of floret number, seed number, seed-setting rate (percentage of seed number in floret number), inflorescence length, inflorescence biomass, seed biomass, reproductive allocation 1 (percentage of inflorescence biomass in ramet biomass), and reproductive allocation 2 (percentage of seed biomass in ramet biomass).

Repeated-measures ANOVA were used to assess the effects of leaf age and growth time on photosynthetic rate and photosynthetic contribution and to assess the effects of organ type and growth time on organ biomass; the results were reported using the Greenhouse–Geisser correction when Mauchly’s test of sphericity was violated. The photosynthetic contribution of individual leaves of reproductive ramets was calculated as follows:

(4) $$PC_{i}\left(\%\right)=\left(P_{i}\times A_{i}\right)/\left[\sum_{i=1}^{3}\left(P_{i}\times A_{i}\right)\right]\times 100$$

where *P*_*i*_ and *A*_*i*_ are the net photosynthetic rate and leaf area of the *i* (1a, 2a, and 3a) leaf of reproductive ramets.

To reveal the rules of change in the sexual reproductive performance with respect to differences in the leaf biomass and total biomass of connected vegetative ramets, the eight variables were regressed based on the difference in the leaf biomass and total biomass of connected vegetative ramets using logarithmic, power, exponential, and linear functions in both years, and the model with the lowest Akaike information criterion (AIC) value from these four was considered the best-fitting model (Sugiura, 1978). The growth time was calculated starting from returning green of *L. chinensis* (April 5, 2017), and the corresponding growth time of seven measurement dates was 51, 58, 65, 72, 79, 86, and 93 days.

## Results

### Growth Characteristics of the Flag Leaf and Functional Leaf

At the seed-filling stage, the leaf length, leaf width and leaf area of both 1a- and 2a-functional leaves of the reproductive ramets were significantly larger than those of the flag leaf. The average leaf areas of the 1a-functional leaf (accounting for 53.0% of the total leaf area) and the 2a-functional leaf (accounting for 38.6% of the total leaf area) were 6.3 times and 4.6 times as large as that of the flag leaf (accounting for 8.4% of the total leaf area), respectively (Table 1). These results showed that the flag leaf was short and narrow, while the functional leaves were long and wide.

**TABLE 1:** Growth characteristics of flag leaves and functional leaves of reproductive ramets in *Leymus chinensis* (means ± SE, *n* = 10).

Leaf age	Length (cm)	Width (cm)	Area (cm^2^)
3a-flag leaf	3.52 ± 0.15c	0.34 ± 0.01b	0.81 ± 0.04c
2a-functional leaf	11.83 ± 0.33b	0.46 ± 0.01a	3.73 ± 0.14b
1a-functional leaf	16.12 ± 0.58a	0.48 ± 0.01a	5.13 ± 0.16a
Total	−	−	9.67 ± 0.27

*Different lowercase letters indicate significant differences (*P* < 0.05) between the leaf ages (a, age).*

### Photosynthetic Rate of the Flag Leaf and Functional Leaf

**FIGURE 2:**
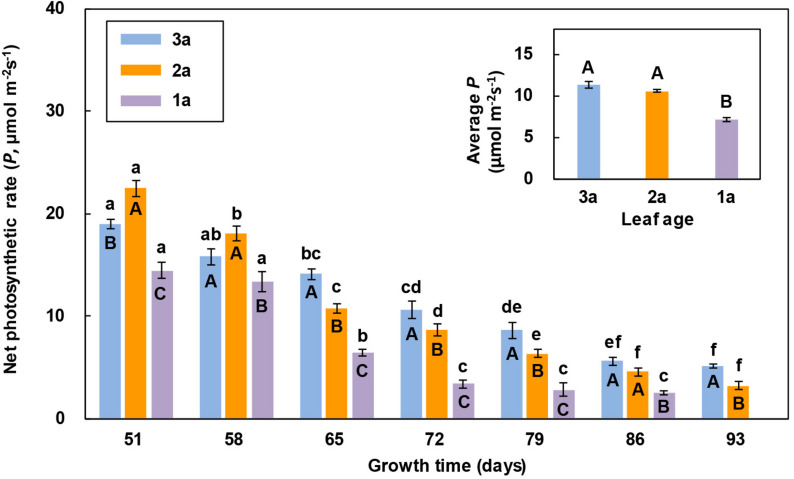
Comparison of the net photosynthetic rate (*P*) of three leaves of reproductive ramets over time (means ± SE, *n* = 10) in *Leymus chinensis*. Different lowercase letters indicate significant differences (*P* < 0.05) between the growth times for the same leaf age; different capital letters indicate significant differences (*P* < 0.05) between the leaf ages for the same growth time. The average *P* of seven growth times is presented as an inset figure. The dates corresponding to the seven growth times are May 26, June 2, June 9, June 16, June 23, June 30, and July 7 in 2017.

The net photosynthetic rate of the 2a-functional leaf of reproductive ramets was higher than that of the other two leaves in the 1st and 2nd measurements, while the net photosynthetic rate of the flag leaf was higher than that of the other two leaves from the 3rd measurement to the end. The net photosynthetic rate of the 1a-functional leaf was always the lowest and decreased early at the last measurement (Figure 2). With the increase in growth time, the net photosynthetic rate of these three leaves showed a decreasing trend, and the net photosynthetic rate of the 1a-functional leaf and flag leaf significantly decreased from the 3rd measurement, while that of the 2a-functional leaf decreased from the 2nd measurement. The average net photosynthetic rate of both the flag leaf and 2a-functional leaf was significantly higher than that of 1a-functional leaf (Figure 2).

#### Photosynthetic Contribution of the Flag Leaf and Functional Leaf to Seed Production

**FIGURE 3:**
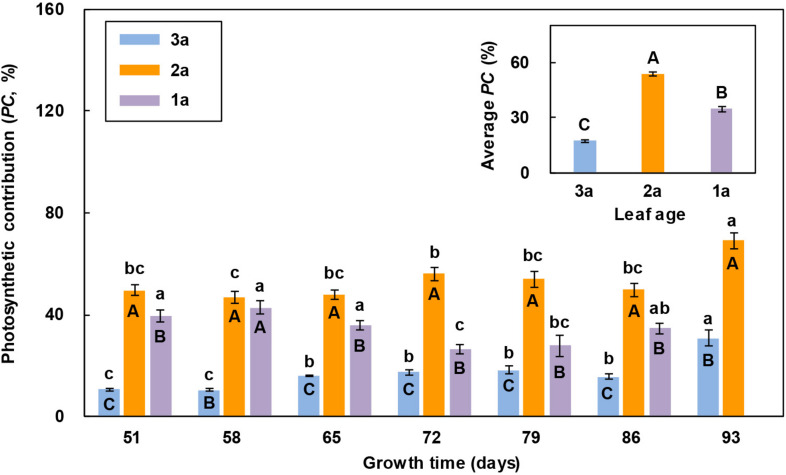
Comparison of the photosynthetic contribution percentage (*PC*) of three leaves of reproductive ramets over time (means ± SE, *n* = 10) in *Leymus chinensis*. Different lowercase letters indicate significant differences (*P* < 0.05) between the growth times for the same leaf age; different capital letters indicate significant differences (*P* < 0.05) between the leaf ages for the same growth time. The average *PC* of seven growth times is presented as an inset figure. The dates corresponding to the seven growth times are May 26, June 2, June 9, June 16, June 23, June 30, and July 7 in 2017.

In all the measurements, the photosynthetic contribution of the 2a-functional leaf to seed production was significantly greater than that of the other two leaves, except that the 1a-functional leaf died prematurely and its photosynthetic contribution decreased to zero in the last measurement (Figure 3). The photosynthetic contributions of the flag leaf and 2a-functional leaf to seed production both showed an increasing trend with the increase in growth time on the whole, while that of the 1a-functional leaf was the greatest in the 2nd measurement and the least in the last measurement. The average photosynthetic contribution to seed production of the 2a-functional leaf was significantly greater than that of the other two leaves, while that of the flag leaf was significantly less than that of the other two leaves (Figure 3).

### Variation in the Organ Biomass of Reproductive Ramets Over Time

**FIGURE 4:**
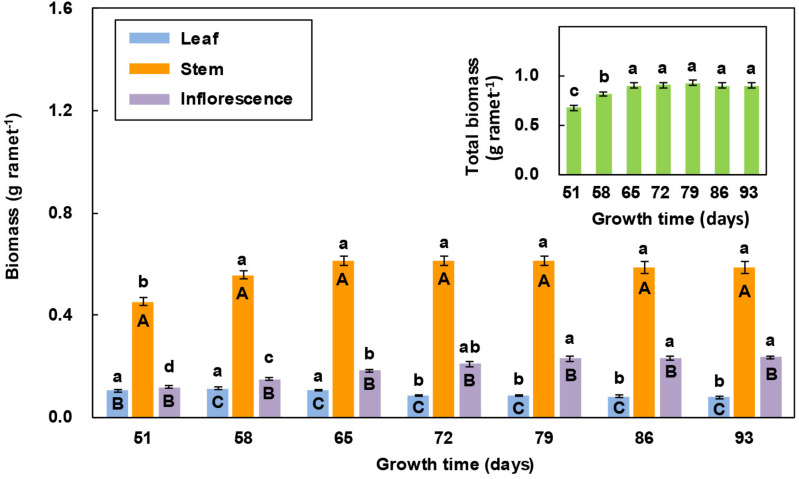
Comparison of the biomass of three organs of reproductive ramets over time (means ± SE, *n* = 10) in *Leymus chinensis*. Different lowercase letters indicate significant differences (*P* < 0.05) between the growth times for the same organ; different capital letters indicate significant differences (*P* < 0.05) between the organ types for the same growth time. The total biomass of three organs is presented as an inset figure. The dates corresponding to the seven growth times are May 26, June 2, June 9, June 16, June 23, June 30, and July 7 in 2017.

In all the measurements, stem biomass was significantly greater than leaf biomass and inflorescence biomass, while leaf biomass was the smallest (Figure 4). With increasing growth time, both leaf biomass and stem biomass first increased and then decreased, while inflorescence biomass showed an increasing trend. For leaf biomass, there was no significant difference between the first three and the last four measurements. Stem biomass significantly increased in the 2nd measurement, but there was no significant difference among the last six measurements. Inflorescence biomass significantly increased in the first three measurements, but there was no significant difference among the last four measurements. The total biomass of individual reproductive ramets significantly increased in the first three measurements and then remained relatively stable (Figure 4).

### Effects of the Number of Vegetative Ramets Connected to Tillering Nodes on Sexual Reproductive Performance

**FIGURE 5:**
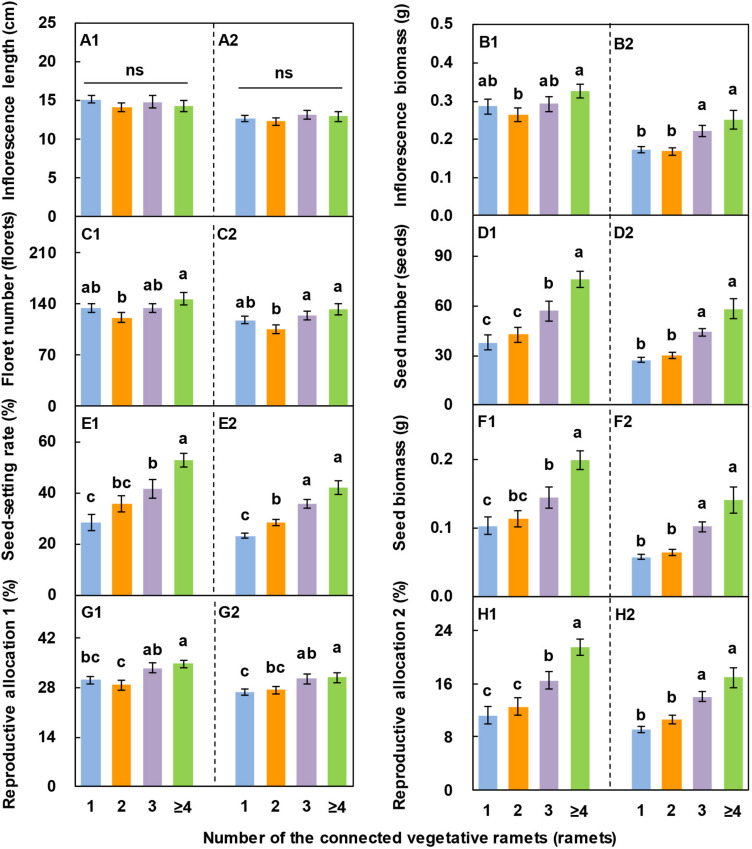
Effects of the number of vegetative ramets connected to tillering nodes on sexual reproductive characteristics in *Leymus chinensis* populations over two consecutive years (2017, **A1–H1**; 2018, **A2–H2**). Data are the means ± SE (*n* = 25). Different lowercase letters indicate significant differences (*P* < 0.05) between different numbers of vegetative ramets, and ns indicates that there is no significant difference (*P* > 0.05) between different numbers of vegetative ramets.

With an increase in the number of vegetative ramets connected to tillering nodes, the inflorescence biomass, floret number, seed number, seed-setting rate, seed biomass, reproductive allocation 1, and reproductive allocation 2 of *L. chinensis* showed an increasing trend on the whole over the two consecutive years (Figures 5B1–H1, B2–H2), whereas the inflorescence length did not (Figures 5A1, A2). The seed number, seed-setting rate, seed biomass, reproductive allocation 1, and reproductive allocation 2 of the reproductive ramets connected to ≥4 vegetative ramets were 1.0 times, 85.6%, 92.2%, 15.3%, and 91.0% higher, respectively, than those of the reproductive ramets connected to one vegetative ramet in 2017 and 1.1 times, 80.9%, 1.4 times, 15.2%, and 87.2% higher, respectively, than those of the reproductive ramets connected to one vegetative ramet in 2018. These results demonstrated that inflorescence length was relatively stable over the two consecutive years, whereas the other seven characteristics fluctuated markedly.

**FIGURE 6:**
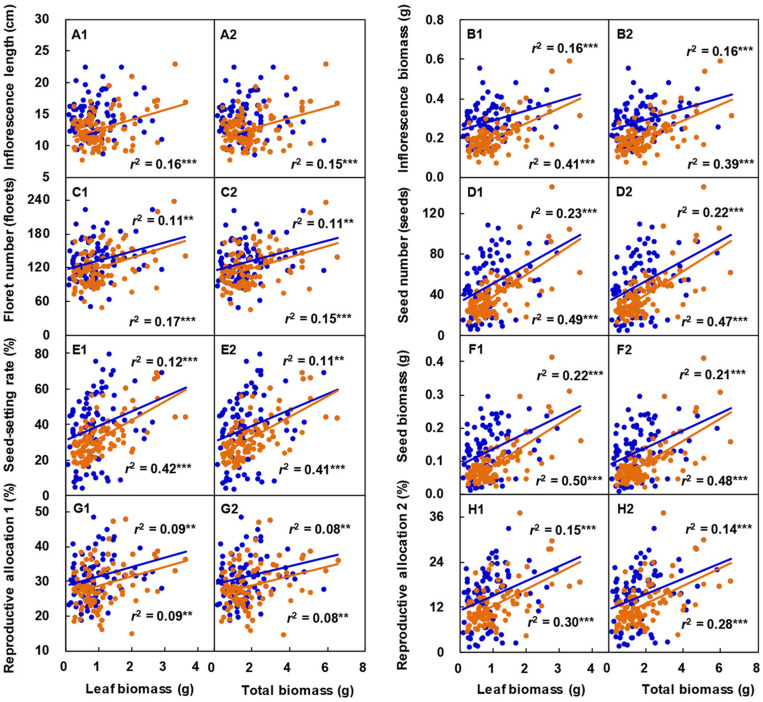
Correlation between the sexual reproductive characteristics and both leaf biomass **(A1–H1)** and total biomass **(A2–H2)** of vegetative ramets connected to tillering nodes in *Leymus chinensis* populations over two consecutive years. The colored circles and lines represent observed data and their fitting lines in 2017 (blue, *n* = 100) and 2018 (orange, *n* = 100). **P < 0.01; ***P < 0.001.

With increasing leaf biomass (Figures 6A1–H1) or total biomass (Figures 6A2–H2) of the vegetative ramets connected to tillering nodes, the inflorescence length, inflorescence biomass, floret number, seed number, seed-setting rate, seed biomass, reproductive allocation 1, and reproductive allocation 2 of the reproductive ramet of *L. chinensis* increased linearly. Except for those involving the inflorescence length in 2017, all other relationships reached a significant level.

### Transfer of ^15^N From Vegetative Ramets to Connected Reproductive Ramets

At the seed-filling stage, the leaf *δ*^15^N, stem *δ*^15^N, and inflorescence *δ*^15^N of reproductive ramets in the ^15^N labeling treatment were all significantly greater than those in the control treatment over the two consecutive years (Table 2). The amount of translocated ^15^N in the stem of reproductive ramets was significantly greater than that in the leaf and inflorescence, and the amount of translocated ^15^N in the inflorescence was the least (Table 3).

**TABLE 2:** Comparison of the *δ*^15^N of reproductive ramets in *Leymus chinensis* between the control and ^15^N labeling treatments (means ± SE, *n* = 4).

Variable	Year	CK	^15^N labeling	*t*	*P*
Leaf *δ*^15^N	2017	1.45 ± 0.53	72.09 ± 4.86	–14.44	0.001
	2018	2.78 ± 0.36	138.06 ± 16.16	–8.37	0.004
Stem *δ*^15^N	2017	0.98 ± 0.57	90.46 ± 10.59	–8.44	0.003
	2018	2.45 ± 0.32	196.67 ± 12.17	–15.95	0.001
Inflorescence *δ*^15^N	2017	1.26 ± 0.37	23.37 ± 1.80	–12.02	< 0.001
	2018	1.88 ± 0.17	41.06 ± 5.12	–7.66	0.005

**TABLE 3:** Mean translocated ^15^N mass allocation to various organs of reproductive ramets in *Leymus chinensis* over two consecutive years (means ± SE, *n* = 4).

Variable	2017		2018	
	Mass (μg)	Percentage (%)	Mass (μg)	Percentage (%)
Leaf ^15^N	0.47 ± 0.05b	14.35 ± 1.79b	0.77 ± 0.08b	14.08 ± 1.57b
Stem ^15^N	2.65 ± 0.23a	79.19 ± 2.16a	4.34 ± 0.17a	78.85 ± 1.14a
Inflorescence ^15^N	0.21 ± 0.02c	6.46 ± 0.82c	0.39 ± 0.03c	7.08 ± 0.60c
Total	3.33 ± 0.21	100	5.50 ± 0.15	100

*Different lowercase letters for each year indicate significant differences (*P*<0.05) between the organs.*

## Discussion

### Photosynthetic Contribution of the Flag Leaf and Functional Leaf to Seed Production

The main driving force for the growth and biomass production of plants is photosynthesis, which provides the carbon and energy required for the synthesis of organic compounds necessary for development (Field et al., 1998; Nowicka et al., 2018). In clonal plant populations, seed production primarily relies on the photosynthetic source-sink relationship. Leaves are primarily involved in photosynthesis and are material producers and exporters. The number and size of leaves determine a plant’s photosynthetic potential and play important roles in determining plant yield, stress responses and disease resistance (Pérez-Pérez et al., 2010; Yang et al., 2015). In the present study, in *L. chinensis* populations growing in a field homogeneous environment, only 6.7% of reproductive ramets had four leaves, and 93.3% had three leaves. Among the reproductive ramets with three leaves, the flag leaf area was significantly smaller than the functional leaf area (Table 1). The average area of the flag leaf (0.81 cm^2^) was only 8.4% of the total leaf area (9.67 cm^2^), reflecting the very small flag leaf of *L. chinensis*. This is very different from the leaf growth characteristics of cereal crops. It has been reported that wheat (*Triticum aestivum*) generally has 6–8 leaves (Berry et al., 1980), and the areas of the top three leaves are approximately the same in size (approximately 20 cm^2^ on average) (Yin et al., 2019); rice has 9–20 leaves (Yin and Kropff, 1996), and the average areas of the flag leaf and the top second leaf are 43.6 and 51.8 cm^2^ (Li et al., 1998). Thus, both the number and size of leaves of *L. chinensis* are evidently less than those of wheat and rice. A large number of studies have confirmed that there is a significant positive correlation between flag leaf area and grain yield (Dhiman et al., 1980; Yue et al., 2006; Sedeek et al., 2009). Therefore, the extremely small leaf area of reproductive ramets may be related to their weak capacity for seed production in *L. chinensis*.

The leaf photosynthetic rate is an important physiological parameter to determine the dry mass production, which reflects the ability of a leaf to assimilate CO_2_. In this study, we found that the net photosynthetic rate of the 2a-functional leaf of reproductive ramets was higher than that of the other two leaves in the first two measurements (Figure 2), indicating that the 2a-functional leaf was the most photosynthetically active and had the strongest ability to assimilate CO_2_ at the heading stage. However, from the 3rd measurement to the end, the net photosynthetic rate of the flag leaf was higher than that of the other two leaves (Figure 2), indicating that the flag leaf was the most photosynthetically active from the flowering to seed maturity stages. When the leaf area was combined with the net photosynthetic rate, the photosynthetic contribution of the flag leaf was always lower than that of the other two functional leaves (Figure 3). This finding does not agree with our first hypothesis, mainly because the flag leaf area of *L. chinensis* is particularly small, which weakens its photosynthetic rate advantage. Our result is inconsistent with the findings in cereal crops such as wheat and rice that photosynthesis of the flag leaf is the primary contributor to grain yield (Walpole and Morgan, 1972; Yoshida, 1972; Araus and Tapia, 1987; Li et al., 1998). This also means that the flag leaf of *L. chinensis* does not have the special mass production function of the flag leaf of cereal crops; instead, the functional leaf is the primary contributor to seed production.

### Senescence of the Flag Leaf and Functional Leaf

The leaf photosynthetic rate is also an important parameter representing leaf vigor (Humbeck et al., 1996; Wingler et al., 1998). In this study, the net photosynthetic rate of the flag leaf and functional leaves of reproductive ramets decreased gradually with increasing growth time from the heading stage (Figure 2), implying that the vigor of the flag leaf and functional leaves gradually declined and natural senescence occurred in all leaves. Senescence is the final stage of leaf development in plants. In leaf senescence, the most obvious outward sign is leaf yellowing, yet the most remarkable inner event is the disintegration of the photosynthetic apparatus in chloroplasts and the degradation of chlorophyll, leading to a concomitant decrease in photosynthetic activity (Woolhouse, 1987; Zheng et al., 2008). However, the senescence rate varies among leaves at different leaf positions on the same plant. Rousseaux et al. (1996) reported that artificial far-red enrichment of the light penetrating through the canopy easily accelerated senescence of the lower rather than the upper leaves in *Helianthus annuus*. When all the leaves of the plant are exposed to the same light conditions, the oldest ones senesce most quickly (Terashima et al., 2005). However, when certain leaves of the plant are shaded, such leaves senesce more quickly (Weaver and Amasino, 2001). We found that the net photosynthetic rate of the flag leaf was higher than that of the other two functional leaves in *L. chinensis* during growth for between 65 and 93 days (Figure 2), showing that the process of leaf senescence was delayed in the flag leaf and much slower than that in the 1a-functional leaf in particular. Some studies have clearly indicated that the difference in the red light to far-red light (R/FR) ratio received by leaves is related to leaf senescence (Anten et al., 2000; Smith, 2000; Terashima et al., 2005). In upright plants, young leaves (i.e., the flag leaf and 2a-functional leaf in this study) shade old leaves (i.e., 1a-functional leaf in this study); therefore, the R/FR ratio received by the old leaves decreases (Terashima et al., 2005), which promotes leaf senescence.

### Allocation of Translocated Resources Between Organs of Reproductive Ramets

Isotope labeling technique is an effective method to explore resources translocation between connected ramets of clonal plants (Zhang et al., 2002). In this study, when the vegetative ramets were labeled with ^15^N at the seed-filling stage in clonal populations of *L. chinensis* growing in a field homogeneous environment, a significantly larger amount of ^15^N than the background value was detected in the connected reproductive ramets over the two consecutive years (Table 2). This result suggests that vegetative ramets can provide the connected reproductive ramets with resources at the most critical stage of sexual reproduction in *L. chinensis*. A study concerning the perennial stoloniferous grass *Agrostis stolonifera* found that at the beginning of daughter establishment, the amount of ^15^N translocated toward daughters was higher than that of ^15^N translocated toward mothers; when daughters reached the size of mothers, the amount of ^15^N translocated toward mothers was higher than that of ^15^N translocated toward daughters, indicating that the amount of translocated ^15^N was significantly affected by the direction of translocation and the developmental status of daughter ramets (Duchoslavová and Jansa, 2018). However, above the study with regard to *A. stolonifera* focuses only on resources translocation between vegetative ramets and vegetative ramets with the same function. Studies on resources translocation between vegetative ramets and reproductive ramets with different functions are still lacking. Thus, it appears that it will be of great significance to carry out dynamic research on bidirectional translocation of resources between different functional ramets in the future.

A study regarding an ornamental plant *Iris laevigata* showed that vegetative ramets could translocate their resources to the connected reproductive ramets (Wang et al., 2017). Our study identified the allocation pattern and strategy of translocated resources between organs of reproductive ramets in *L. chinensis*. We found that the amount of translocated ^15^N was the largest in the stem, followed by the leaf, and it was the lowest in inflorescence (Table 3). Simultaneously, we also found that from the heading stage, all leaves of reproductive ramets gradually senesced (Figure 2), inflorescence biomass persistently increased, leaf biomass and stem biomass slightly decreased in late reproductive growth, but they remained statistically stable for a long time (leaf biomass remained stable between the 4th and 7th measurements, and stem biomass remained stable between the 2nd and 7th measurements) (Figure 4). All of these results suggest that with the senescence of leaves in physiological function, not only the stem but also the leaves (assimilating organs) require an external resource supply to maintain the metabolic consumption of life-sustaining activities and to maintain the physiological functions of leaves. This finding means that the amounts of translocated resources allocated to the vegetative organs (stem and leaf) for life-sustaining activities are much larger than those allocated to the reproductive organs (inflorescence) for forming seeds. Therefore, at least from the perspective of a short-term resource transfer, reproductive ramets adopt a strategy of growth first and reproduction later to allocate the translocated resources between organs.

### Role of Vegetative Ramets Connected to Tillering Nodes in Sexual Reproduction

In clonal grass populations, it is often seen that a reproductive ramet has different numbers of connecting vegetative ramets on its tillering nodes. This is possibly because there are differences in size of reproductive ramets, capacity for vegetative propagation of tillering nodes of reproductive ramets, or environmental conditions (Xiao et al., 2011; Yuan et al., 2020). In this study, under a field homogeneous condition, we selected reproductive ramets of a similar size and with different numbers of connecting vegetative ramets at the edge of each plot where there was no competition between ramets, to carry out the experiment. Therefore, the difference in the number of connecting vegetative ramets involved in this study more likely result from the difference in capacity for vegetative propagation of tillering nodes of reproductive ramets and consequently the number of axillary buds produced.

In this study, at the early heading stage, we selected reproductive ramets of a similar size, with synchronous heading, and with different numbers of connecting vegetative ramets, as the experimental samples, thus reducing or eliminating the impacts of inherent difference and asynchronous heading on sexual reproduction performance (Li et al., 2018). Additionally, the selected experimental samples were all located at the edge of each plot. Considering the small density of ramets at the edge, the large interval between ramets, and the proximity of resource supply, compared with vegetative ramets on the tillering nodes, other ramets had tiny effects on sexual reproduction performance. All ramets of *L. chinensis* in the experimental plots originally came from the same natural clone and were genetically identical, thereby excluding the effect of genetic differences on sexual reproduction performance. Therefore, we were pretty certain that vegetative ramets connected to tillering nodes had positive effects on the inflorescence biomass, floret number, seed number, seed-setting rate, seed biomass, reproductive allocation 1, and reproductive allocation 2 (Figures 5B1–H1, B2–H2), and these parameters were significantly and positively correlated with the biomass of the vegetative ramets (Figure 6). These results are against the second hypothesis. Most importantly, we showed that vegetative ramets are very advantageous for sexual reproduction under the tillerning node connection form in *L. chinensis*. Our study implies that vegetative ramets play an important role in the sexual reproduction apart from the spatial expansion of clonal plant populations.

Theoretically speaking, the more vegetative ramets connected to the reproductive ramet is, the better the sexual reproductive performance is. However, there are actually some deviations from this trend. We found that the inflorescence biomass and floret number were lowest when the reproductive ramet was connected to two vegetative ramets rather than one vegetative ramet (Figures 5B,C). Under natural conditions, individual sizes are irregular in most plant populations due to change in growth rates caused by factors such as genetic differences, spatial heterogeneity of resources or age differences (Weiner and Solbrig, 1984). Even in clonal populations of *L. chinensis* growing under a homogeneous condition, there were differences in age between the vegetative ramets connected to tillering nodes, leading to an irregularity in size. Therefore, the resource supply of the two connected vegetative ramets may be lower than that of the one connected vegetative ramet, and thus, sexual reproductive performance might also follow such a rule.

The degree to which vegetative ramets affect sexual reproductive performance varies with reproductive characteristics. We found that the number of vegetative ramets had no effect on inflorescence length but had significant effects on seed-related characteristics such as seed number, seed biomass, seed-setting rate, and reproductive allocation 2 (Figure 5), which may be due to the different formation times of each characteristic. On the Songnen Plain, *L. chinensis* usually enters the young spike differentiation stage soon after returning green on April 5, the inflorescence length begins to elongate from the early and medium stage of young spike differentiation and stops growing before the flowering stage (mid-June), and the seeds do not mature until mid to late July (Zhu, 2004). Thus, it appears that the seed-related characteristics, because of their relatively long formation time, will receive resources from the connected vegetative ramets for a relatively long time and thus be strongly affected.

## Conclusion

In clonal populations of *L. chinensis* growing under a field homogeneous condition, the photosynthetic contribution of the functional leaf to seed production was larger than that of the flag leaf; from the heading stage, all leaves of reproductive ramets gradually senesced. Vegetative ramets could translocate their own resources to the connected reproductive ramets through tillering nodes, and a larger proportion of these resources were allocated to vegetative organs to sustain life activities and less allocated to reproductive organs to form seeds. The number of vegetative ramets connected to tillering nodes positively affected sexual reproductive performance. However, the current study was conducted in a field homogeneous environment with only one level of resource supply. Further studies that explore effects of the flag leaf and the number of vegetative ramets connected to tillering nodes on sexual reproductive performance with different levels of resource supply will help us to better understand population adaptation and resource allocation of clonal grasses in future environments.

## Data Availability Statement

The datasets generated for this study are available on request to the corresponding author.

## Author Contributions

HL and YY designed the study. JG collected the data. JG, HL, CZ, and YY analyzed the data and wrote the manuscript. All authors read and approved the final manuscript.

## Conflict of Interest

The authors declare that the research was conducted in the absence of any commercial or financial relationships that could be construed as a potential conflict of interest.
